# Tropical Andean Forests Are Highly Susceptible to Nutrient Inputs—Rapid Effects of Experimental N and P Addition to an Ecuadorian Montane Forest

**DOI:** 10.1371/journal.pone.0047128

**Published:** 2012-10-10

**Authors:** Jürgen Homeier, Dietrich Hertel, Tessa Camenzind, Nixon L. Cumbicus, Mark Maraun, Guntars O. Martinson, L. Nohemy Poma, Matthias C. Rillig, Dorothee Sandmann, Stefan Scheu, Edzo Veldkamp, Wolfgang Wilcke, Hans Wullaert, Christoph Leuschner

**Affiliations:** 1 Albrecht von Haller Institute of Plant Sciences, Georg August University Göttingen, Göttingen, Germany; 2 Institute of Biology, Freie Universität Berlin, Berlin, Germany; 3 Instituto de Ecología, Universidad Técnica Particular de Loja, San Cayetano Alto, Loja, Ecuador; 4 J.F. Blumenbach Intitute of Zoology and Anthropology, Georg August University Göttingen, Göttingen, Germany; 5 Max Planck Institute for Terrestrial Microbiology, Marburg, Germany; 6 Buesgen Institute - Soil Science of Tropical and Subtropical Ecosystems, Georg August University Göttingen, Göttingen, Germany; 7 Universidad National de Loja, Ciudadela Universitaria Guillermo Falconí sector La Argelia, Loja, Ecuador; 8 Geographic Institute, University of Berne, Berne, Switzerland; 9 Geographic Institute, University of Mainz, Mainz, Germany; Lakehead University, Canada

## Abstract

Tropical regions are facing increasing atmospheric inputs of nutrients, which will have unknown consequences for the structure and functioning of these systems. Here, we show that Neotropical montane rainforests respond rapidly to moderate additions of N (50 kg ha^−1^ yr^−1^) and P (10 kg ha^−1^ yr^−1^). Monitoring of nutrient fluxes demonstrated that the majority of added nutrients remained in the system, in either soil or vegetation. N and P additions led to not only an increase in foliar N and P concentrations, but also altered soil microbial biomass, standing fine root biomass, stem growth, and litterfall. The different effects suggest that trees are primarily limited by P, whereas some processes—notably aboveground productivity—are limited by both N and P. Highly variable and partly contrasting responses of different tree species suggest marked changes in species composition and diversity of these forests by nutrient inputs in the long term. The unexpectedly fast response of the ecosystem to moderate nutrient additions suggests high vulnerability of tropical montane forests to the expected increase in nutrient inputs.

## Introduction

Since the 1950/60s, anthropogenic changes to the cycling of the key nutrients nitrogen (N) and phosphorus (P) have dramatically altered the structure and functioning of many ecosystems in the world's industrialized regions [1]–[6]. Elevated N and P inputs affect virtually all components and processes of terrestrial and aquatic ecosystems, including plant growth, plant longevity and stress tolerance, plant community composition and diversity, biotic interactions (plant-plant, plant-fungus, plant-animal), the composition and activity of heterotrophic communities, and the storage and cycling of carbon, nutrients and water [7]–[9]. This is because primary production is limited by N or P, or both in the vast majority of ecosystems around the globe [8], [10]–[14].

In the past 30 years, research has focused on the structural and functional responses of temperate and boreal forests to atmospheric N inputs [15]–[18] because the bulk of fertilizer use worldwide was in the industrialized nations of the northern hemisphere. Furthermore, these regions had particularly pronounced gaseous NO_x_ emissions originating from the combustion of fossil fuels and NH_3_ emissions from animal production [1]. However, this situation is changing rapidly. With the expansion of industrial agriculture into many tropical and southern hemispheric regions, the spread of N and P compounds to adjacent and more distant non-agricultural ecosystems in these regions has been greatly increased [1], [19]–[20]. In the future, tropical forests will be increasingly exposed to airborne N and P inputs. Higher P inputs are mostly due to the deposition of dust [4], [21]–[24], but sources of N can be varied, including oxidised and reduced N compounds emitted with farming, livestock breeding and the combustion of fossil fuels, and N released through biomass burning with the conversion of tropical forests [1], [25]–[26].

Although tropical forests are likely to be sensitive to these changes, the size and direction of their responses are unclear [7], [20], [27]–[31]. A number of fertilization experiments in tropical forests have investigated responses to experimental high-dose treatments with N, or N and P (100–300 kg N ha^−1^ yr^−1^ and/or 50–100 kg P ha^−1^ yr^−1^) [30], [32]–[40]. These experiments typically focused on selected ecosystem properties, such as changes in tree growth, fine litter production or soil carbon pools, but did not provide comprehensive insight into ecosystem responses to elevated N and P loads. Here, we report data from a nutrient manipulation experiment (NUMEX) investigating the response of an old-growth montane forest ecosystem in the Andes of southern Ecuador to moderate N (50 kg ha^−1^ yr^−1^) and/or P (10 kg ha^−1^ yr^−1^) additions considering a multitude of response variables. The study allows comprehensive insight into how highly diverse tropical montane forest ecosystems respond to moderate nutrient additions such as those predicted by climate change scenarios.

## Results and Discussion

A large number of ecosystem properties and functions exhibited marked responses to the experimental nutrient additions after only one year.

### Soil nutrient pools and soil biological activity

**Figure 1:**
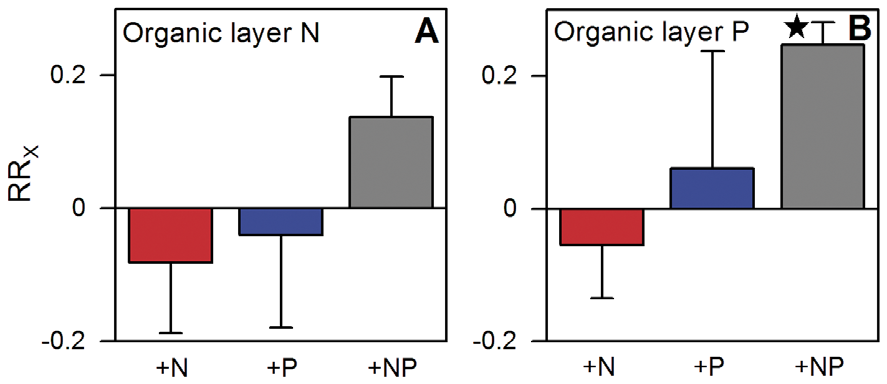
Effects of one year of experimental nutrient addition on various soil nutrient pools of a montane forest in Ecuador. Effects are presented as natural-log transformed response ratios (RR_X_) in which the parameter in the enriched treatment is divided by its value in the control treatment and then ln-transformed. Hence, a value of 0.2 indicates a value in the manipulated treatment that is c. 23% higher than in the control, while a value of 0.5 indicates a 65% increase. Error bars indicate plus or minus one standard error. Data of the control treatment (mean ±1 SE) are given in parentheses below. Asterisks indicate significant differences to the control (P≤0.05). a. Organic layer nitrogen pool (3.79±0.31 Mg N ha^−1^). b. Organic layer phosphorus pool (98.6±6.8 kg P ha^−1^).

**Figure 2 pone-0047128-g002:**
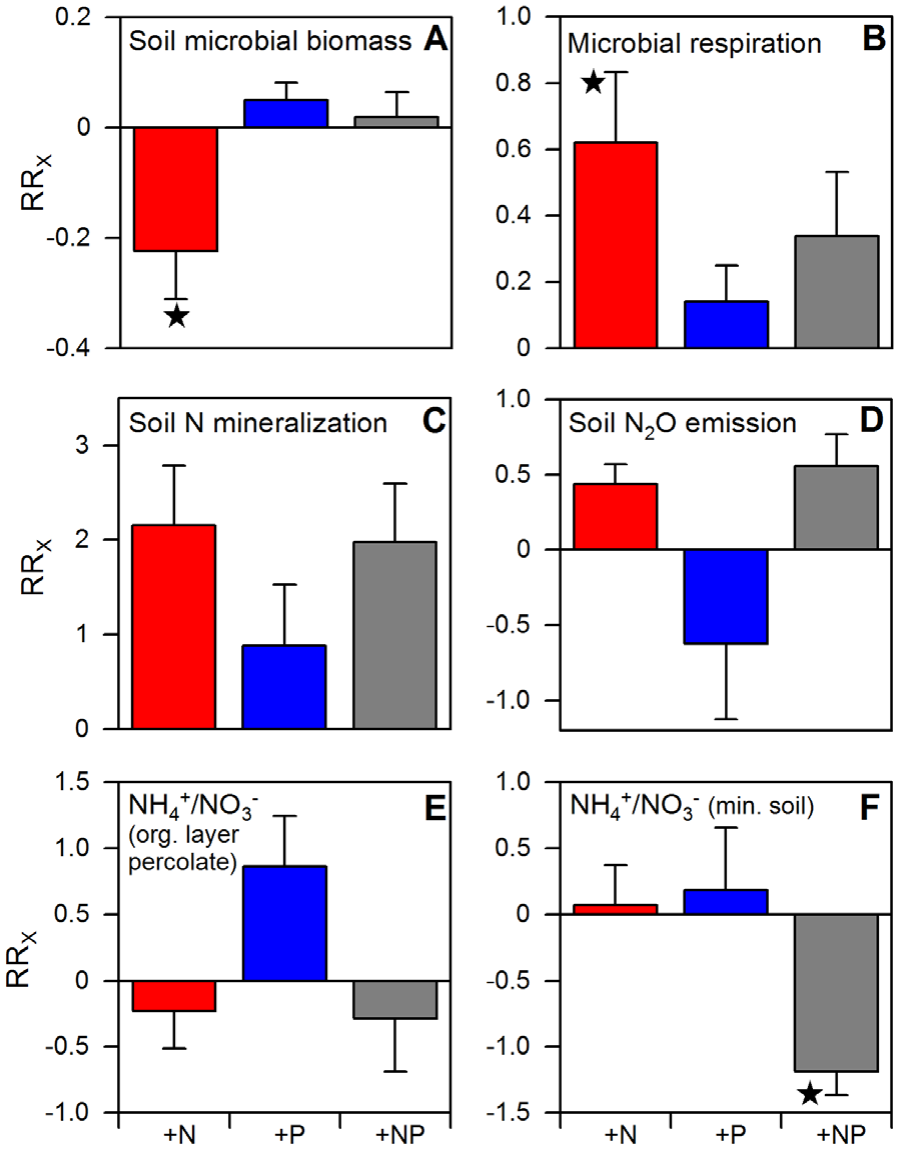
Effects of one year of experimental nutrient addition on biological soil activity of a montane forest in Ecuador. a. Soil microbial biomass in May 2009 (5881±25 µg C_mic_ g^−1^ soil dry mass). b. Respiration of soil microorganisms in May 2009 (5.066±0.36 µl O_2_ mg C_mic_^−1^ h^−1^). c. Net N mineralization in September 2008 (23.4±10.5 ng N cm^2^ h^−1^). d. Annual emission of N_2_O (0.25±0.03 kg N ha^−1^ yr^−1^). e. Mean NH_4_^+^/NO_3_^−^ ratio of organic layer percolate from February 2008 to January 2009 (15.7±6.7) and f. mean NH_4_^+^/NO_3_^−^ ratio of mineral soil solution from February 2008 to January 2009 (8.9±2.0). Error bars indicate plus or minus one standard error. Data of the control treatment (mean ±1 SE) are given in parentheses in the legend. Asterisks indicate significant differences to the control (P≤0.05). For interpretation of graph see legend of Fig. 1.

Nutrient addition did not result in a significant increase of the organic layer N pool while the P pool increased after combined addition of N and P (Fig. 1). The reason is likely the relatively high N stock in the thick organic layer. An adjacent micro-catchment between 1850 and 2150 m a.s.l. had 0.9–21 Mg ha^−1^ N in the organic layer while the P stock ranged from 30–700 kg ha^−1^ [41]. Soil microbial biomass decreased after adding N (Fig. 2a), whereas P addition had no effect. A decrease in microbial biomass after N addition suggests detrimental effects of nitrogen on soil microorganisms and has been seen in other studies [3], [42], in particular with lignin decomposing fungi [43]–[44]. Conforming to the assumption of detrimental effects on microorganisms using complex organic compounds the metabolic activity of microorganisms significantly increased after N addition (Fig. 2b), indicating a shift towards microorganisms predominantly using more easily available carbon resources.

All treatments resulted in slightly higher net N mineralization rates, the increase after N addition was marginally significant (p = 0.08, Fig. 2c). N_2_O emissions tended to increase after N+P addition (p = 0.05) but not after addition of N or P only (Fig. 2d). This suggests that N is needed as substrate for denitrification while P is limiting the respective organisms.

The assumption that P addition stimulated microorganisms which are responsible for N transformations is further supported by the finding that the combined addition of N and P had a significant effect on the NH_4_-N/NO_3_-N ratio in mineral soil solution (Fig. 2e–f). Although we added urea, which is primarily a source of NH_4_^+^, NO_3_^−^ concentrations increased after combined N and P addition while NH_4_^+^ concentrations did not. This was probably because of the combination of stimulated nitrification and microbial NH_4_^+^ retention. Our finding that only N and P addition together stimulated N mineralization (and possibly also nitrification) is different than reports from other tropical sites where N addition alone stimulated nitrification and triggered NO_3_^−^ losses to the subsoil. This difference may be the result of no - or less pronounced - P limitation of the involved microorganisms [27], [45]. In contrast to many temperate forests where NO_3_^−^ is the most abundant N form [46], NO_3_^−^ only accounted for 3–5% of total N in the soil solutions from our study site. Instead, the soil N pool was predominantly made up of dissolved organic nitrogen (DON) and NH_4_^+^ (contributing 50–70% and 27–43% respectively) [47]. This implies that small changes in N mineralization and nitrification rates can have a large impact on NO_3_^−^ concentrations and fluxes. Our results indicate that the addition of different nutrients may stimulate or inhibit different processes in the ecosystem resulting in a complex system response to nutrient deposition.

### Nutrient cycling

**Figure 3 pone-0047128-g003:**
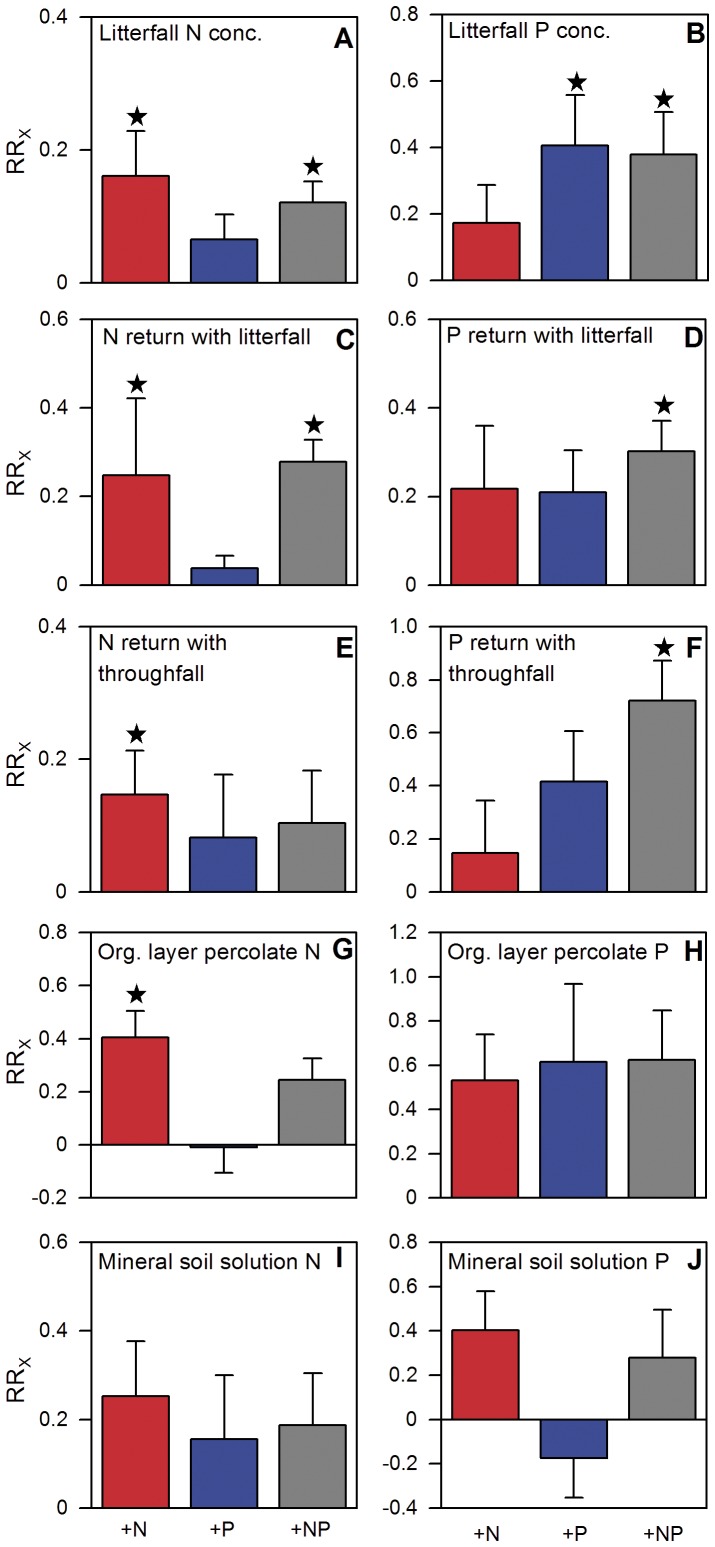
Effects of one year of experimental nutrient addition on nutrient cycling of a montane forest in Ecuador. a. Nitrogen and b. phosphorus concentrations of the litterfall in January 2009 after one year of nutrient addition (0.87±0.03% N and 0.025±0.006% P). Total annual return of c. nitrogen and d. phosphorus with litterfall (41.7±5.0 kg N ha^−1^ yr^−1^ and 1.49±0.24 kg P ha^−1^ yr^−1^). Total annual return of e. nitrogen and f. phosphorus with throughfall (10.1±1.0 kg N ha^−1^ yr^−1^ and 0.15±0.03 kg P ha^−1^ yr^−1^). g. Annual flux of nitrogen and h. phosphorus found in the organic layer percolate (14.65±1.21 kg N ha^−1^ yr^−1^ and 0.13±0.02 kg P ha^−1^ yr^−1^). i. Annual flux of nitrogen and j. phosphorus found in the soil solution at 0.3 m soil depth (3.26±0.59 kg N ha^−1^ yr^−1^ and 0.03±0.004 kg P ha^−1^ yr^−1^). Error bars indicate plus or minus one standard error. Data of the control treatment (mean ±1 SE) are given in parentheses in the legend. Asterisks indicate significant differences to the control (P≤0.05). For interpretation of graph see legend of Fig. 1.

Increased N and P contents in litterfall and throughfall (Figs. 3a–f) indicate that a large proportion of the added nutrients was taken up by trees and subsequently accelerated nutrient cycling through higher N and P return with litterfall and leachate [48] and through stimulated litter decomposition by higher N and P concentrations [49]. The annual increase in N and P fluxes in litterfall and throughfall after fertilization was equivalent to 25.4% and 26.7% of the applied N, after N addition and after N and P addition, respectively, and 3.8% and 6.1% of the applied P, after P addition and after N and P addition, respectively. Neither organic layer N and P pools nor N and P losses to the atmosphere or to the subsoil were significantly increased by N or P addition (Figs. 2d and 3i, j), suggesting that the bulk of N and P added was retained in the ecosystem [48]. The N and P effects on nutrient cycling were interrelated. Phosphorus increased the retention of N in the system since aboveground N losses were slightly reduced (Fig. 2d) and losses by leaching were negligible (Fig. 3i–j). We attribute this mainly to the stimulation of the N-mineralizing microbial community by P addition as reflected by the significantly increased net N mineralization rates [50]. Furthermore, N application increased the P return with litterfall and throughfall when added in combination with P (N+P treatment: Figs. 3d,f). The positve effects of N addition on litter P concentration and P return with litterfall and throughfall might be a result of better soil P availability to plants by increased extracellular phosphatase activity after N addition. This has been observed in several temperate and tropical forest studies [51]–[53].

### Tree biomass and forest productivity

**Figure 4 pone-0047128-g004:**
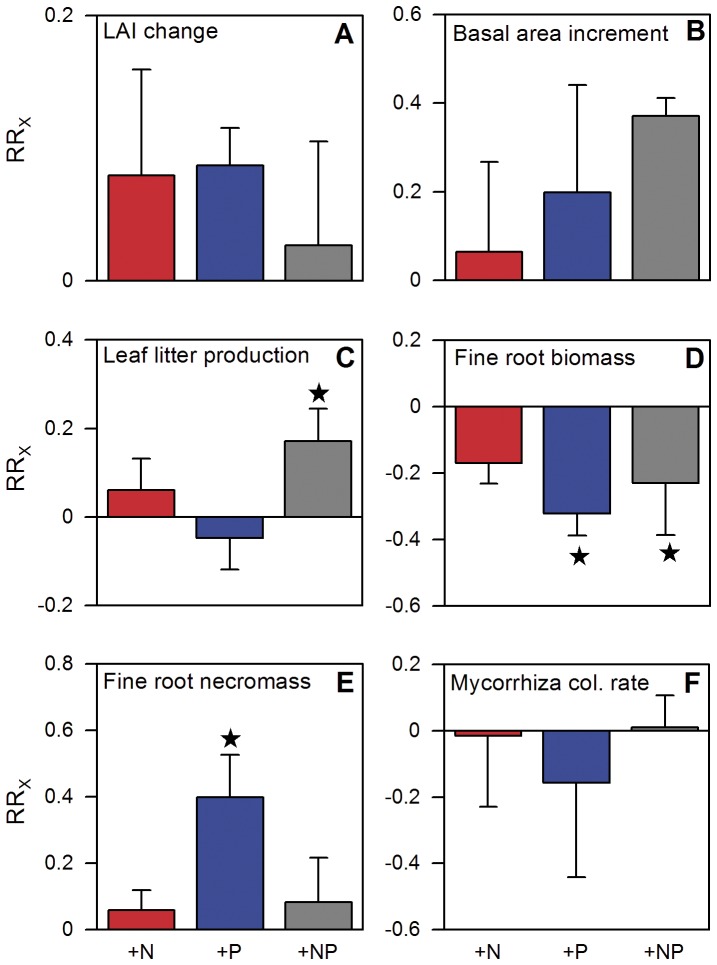
Effects of one year of experimental nutrient addition on vegetation related parameters of a montane forest in Ecuador. a. Relative change of leaf area index (LAI) after one year of nutrient addition (measurements from January 2009 were compared to measurements prior to nutrient addition in January 2008; control mean changed from 4.6±0.2 in 2008 to 4.7±0.4 in 2009). b. Plot basal area increment from February 2008 to January 2009 (0.111±0.018 m^2^ ha^−1^). c. Annual leaf litter production from February 2008 to January 2009 (3.46±0.46 Mg ha^−1^). d. Fine root biomass in January 2009 (443±28 g m^−2^), e. fine root necromass in January 2009 (426±29 g m^−2^), and f. rate of fine root colonization by arbuscular mycorrhizal fungi in January 2009 (53.3±6.2%). Error bars indicate plus or minus one standard error. Data of the control treatment (mean ±1 SE) are given in parentheses in the legend. Asterisks indicate significant differences to the control (P≤0.05). For interpretation of graph see legend of Fig. 1.

Stand leaf area index (LAI) tended to increase after addition of N and P alone (although not significantly), while leaf litter production increased only after N+P addition (Figs. 4a,c). Since N fertilization tended to increase the specific leaf areas and foliar N concentrations of the four most common tree species (Table 1; difference significant only for *Myrcia* sp.), two foliage attributes generally associated with a shorter leaf lifespan [54], we infer that N addition has stimulated leaf production. The LAI increase after P addition probably resulted from an extension of mean leaf lifespan as indicated by the observed slight reduction in litter production. This is supported by results from Hawaiian montane forests, where leaf production and leaf longevity were increased after P addition [55].

**Table 1:** Effects of nutrient addition on foliar nutrient concentrations and leaf morphology of the four most common tree species of a montane forest in Ecuador.

			Deviation from control (%)		
	Tree species	control	+N	+P	+NP
Foliar N (mg g^−1^)	*Graffenrieda*	12.2	+5	±0	+4
	*Myrcia* sp.	11.3	+9^*^	−6	+11^*^
	*Hieronyma*	13.8	+1	−4	+11^*^
	*Alchornea*	13.8	+7	+15^*^	+14^*^
Foliar P (mg g−1)	*Graffenrieda*	0.43	−2	+37^*^	+21
	*Myrcia* sp.	0.43	+28^*^	−5	+33^*^
	*Hieronyma*	0.54	+2	+11	+28^*^
	*Alchornea*	0.71	−11	+34^*^	+42^*^
Foliar N/P ratio	*Graffenrieda*	31	+7	−31^*^	−17
	*Myrcia* sp.	27	−17	−4	−18
	*Hieronyma*	26	−3	−14^*^	−14^*^
	*Alchornea*	20	+16^*^	−15^*^	−21^*^
Leaf area (cm^2^)	*Graffenrieda*	178.2	+28	−1	+15
	*Myrcia* sp.	17.9	+3	−15	+12
	*Hieronyma*	26.8	−13	−29	−23
	*Alchornea*	30.5	−8	+1	+2
Specific leaf area (cm^2^ g^−1^)	*Graffenrieda*	38.4	+9	+7	+10
	*Myrcia* sp.	40.2	+2	−5	+2
	*Hieronyma*	69.3	+6	+1	−7
	*Alchornea*	40.9	+13	+5	+12

Given are the absolute values for the control treatment and the percental effects of the treatments. Asterisks indicate significant differences to control (P<0.05). The number of sampled trees was for *Graffenrieda emarginata*: 5 (control), 6 (+N), 6 (+P) and 6 (+NP), for *Myrcia sp.*: 6, 5, 5 and 6, for *Hieronyma fendleri* 2, 5, 5 and 3 and for *Alchornea lojaensis*: 5, 4, 5 and 4.

Stand basal area increment as a proxy of aboveground productivity tended to increase in all fertilization treatments (Fig. 4b), as reported from other Neotropical montane forests after addition of N [34], [39], [55], P [34], [39] or N and P [35].

All fertilizer treatments resulted in a marked reduction in standing tree fine root biomass (by 15–28%) with the effect being strongest after P addition (Fig. 4d). This treatment also led to a strong increase in standing fine root necromass, while the N and N+P treatments had no effect on necromass (Fig. 4e). Presumably, the accumulation of fine root necromass in the P treatment resulted from reduced root litter decay due to an unfavorable litter N∶P ratio for decomposers [56]. A decline of fine root biomass and a concurrent increase of dead roots after nutrient addition have also been shown in other montane forests after N addition [57: Puerto Rico] or after N, P and N+P addition [58: Hawaii].

The observed decrease of fine root biomass does not necessarily indicate a reduced fine root production. More likely is a stimulation of fine root production by nutrient addition and a concurrent increase of fine root turnover [59], [60], the combination of both effects could result in a reduced standing fine root biomass.

The root colonization by arbuscular mycorrhizal fungi (AMF) was not significantly affected by nutrient addition, with values remaining more or less constant at about 50% (Fig. 4f). This result contrasts with the rapid response of fine root biomass and does not fit to the predictions of the functional equilibrium model of AMF [61]. Empirical data show root colonization by AMF to be reduced when N and particularly P availability is increased [62]. These results indicate that in our study area the addition of P (and to a lesser extent of N) relaxed the growth limitation imposed by P (and N) scarcity and prompted the trees to allocate more carbon into aboveground structures and productivity. Our finding that fine root biomass was significantly reduced upon N and P addition, while the mycorrhizal infection of the trees remained unchanged, points at the importance of AMF functions other than nutrient acquisition in controlling the plant-fungus interaction [63], or resource uptake rates that are independent of infection rate.

**Figure 5:**
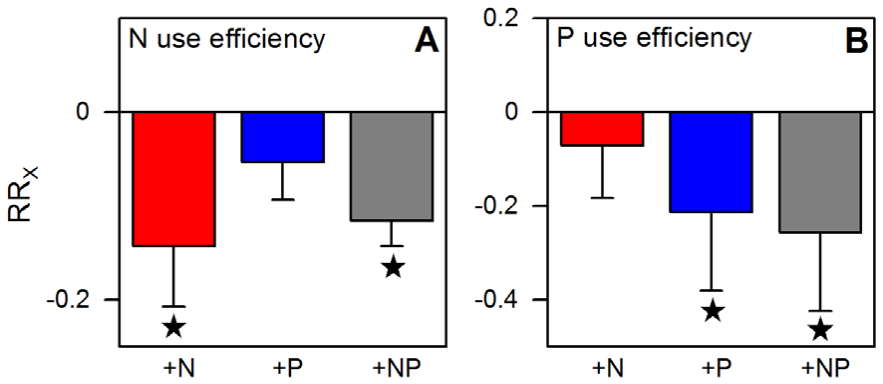
Nutrient use efficiencies and monthly nutrient return with litterfall. Nutrient use efficiencies (i.e. the ratio of total litterfall dry mass to nutrient content [64]) of the different treatments in the studied montane forest in Ecuador after one year of nutrient addition (samples from January 2009, means of N = 24 litter traps per treatment). a. N use efficiency (116.5±3.8 g g^−1^), b. P use efficiency (4751±782 g g^−1^). Error bars indicate plus or minus one standard error. Data of the control treatment (mean ±1 SE) are given in parentheses in the legend. Asterisks indicate significant differences to the control (P≤0.05). For interpretation of graph see legend of Fig. 1.

The stand-level N and P use efficiencies in the control plots were in the upper range of values reported from other tropical forests [64] (Fig. 5). Addition of nitrogen, phosphorus, or both, led to a significant decrease (10–25%) in the N or P use efficiencies, respectively, within one year, suggesting rapid relaxation from growth limitation by P and N and most likely decreased nutrient resorption efficiencies of the vegetation. Lower nutrient resorption efficiencies with increasing green leaf nutrient status were also reported by Kobe et al. [65] for a global data set of perennial plant species.

### Divergent tree species growth responses

We found a highly variable response of stem diameter growth upon N and/or P addition among the tree species in the fertilized plots. Depending on species, growth rates were either higher or lower relative to the control plots (Table 2). However, two of the most common species (*Hieronyma fendleri* and *Alchornea lojaensis*) showed increases in stem diameter growth rates after addition of N, P or N+P, while two other species (*Graffenrieda emarginata* and *Myrcia* sp.) tended to reduce growth upon N or P fertilization. The divergent growth response of different tree species is in agreement with fertilization studies from other tropical montane forests [33], [34]. It appears that the responsiveness to N or P addition of stem diameter growth is highly species-specific and that while some species will increase, others will reduce their competitive strength with continued nutrient addition, likely resulting in species composition changes and diversity reductions in this species-rich forest over time [7], [66]–[67]. Since the forest canopy will become denser in response to fertilization (increasing the LAI) and the trees will be relieved of growth constraints due to limited N and/or P availability, light competition will become more important with increasing input of nutrients. These changes may reduce the competitive ability of the seedlings and saplings of the currently abundant tree species, and will probably result in their eventual replacement by species adapted to more fertile soils. Changes in tree species composition (from slow-growing species adapted to nutrient-poor soils to faster growing species adapted to more fertile soils) will most likely accelerate the projected shifts in the C cycle by increasing the biomass turnover rate. The N∶P ratio in leaf biomass of the unfertilized trees (means of 20–31 in the four most common species) provides support for the conclusion that tree growth in the studied forest is mainly limited by P [68]. Consequently, P was accumulated to a larger extent in the foliage than N after addition of P or N, and the N∶P ratio responded more to P addition (decrease) than to N addition (no uniform effect; Table 1). The increased foliar N concentrations in three of the four studied common tree species after addition of N or N+P, respectively, should result in higher photosynthetic carbon gain, since photosynthetic capacity is closely related to foliar N [69].

**Table 2:** Effects of nutrient addition on annual stem diameter growth of the four most common tree species of a montane forest in Ecuador.

		Deviation from control (%)		
**Tree species**	**control**	**+N**	**+P**	**+NP**
	**(mm)**			
*Graffenrieda emarginata*	0.84 (49)	−12 (49)	−21 (47)	+16 (54)
*Myrcia* sp.	0.80 (17)	−39 (15)	−50^*^ (16)	−5 (11)
*Hieronyma fendleri*	0.07 (6)	+157 (22)	+71 (13)	+414* (13)
*Alchornea lojaensis*	0.12 (20)	+33* (18)	+17 (16)	+42* (9)
all other species pooled	0.28 (78)	−11 (94)	+82 (77)	+25 (89)

Given are the absolute values for the control treatment (February 2008–January 2009) and the percental effects of the treatments. Asterisks indicate significant differences to the control (P<0.05).

### Conclusions

Overall, the strong and complex short-term response of the tropical montane forest ecosystem to moderate nutrient inputs suggests major consequences of expected future nutrient inputs into these ecosystems. This is particularly evident at our study site. The effects that we observed were larger than those reported from tropical lowland forests on more fertile soils, where only long-term nutrient addition resulted in significant effects [40]. Several of the responses to nutrient addition are similar to those known from other tropical montane forests, where they occurred either after chronic nutrient addition or after fertilization with higher amounts of N.

Provided that these initial trends persist, continued addition of substantial amounts of N and P will probably result in taller forests with a higher above-ground biomass but smaller below-ground biomass [60]. However, the below-ground response of the system to nutrient addition is still poorly understood. Given the large stocks of carbon in the organic layer, stimulated mineralization and soil respiration rates and less belowground C sequestration may turn these ecosystems into a significant future source of CO_2_ to the atmosphere.

Further studies have to show how nutrient cycles and key ecosystem services such as carbon storage will adjust to continuing input of moderate amounts of N and P and how community composition will change in the long run.

Cross-study comparisons of nutrient manipulation experiments could contribute to a better understanding of ecosystem responses to increasing nutrient deposition, but the currently published studies are hard to compare due to different levels of fertilizer addition and methodological differences among the various studies. A network of coordinated experiments adding low amounts of nutrients to tropical forests, that covers a wide range of environmental conditions (climate, soil), would be the method choice of obtaining general patterns of tropical forest ecosystem responses to increasing nutrient availability.

## Materials and Methods

### Study area

The study was conducted at about 2000 m elevation, in a tropical montane moist forest of the San Francisco Reserve in the Andes of southern Ecuador (3°58′S, 79°04′W) (Fig. S1). This forest is in nearly pristine condition, and is one of the best-studied tropical montane forests worldwide, known for its extraordinary richness in tree species as well as other plant and animal groups [70]–[71]. The forest harbors more than 300 tree species with Lauraceae, Melastomataceae and Rubiaceae being the plant families with the highest species numbers. The study site has a mean annual precipitation of ∼2200 mm and an annual mean temperature of ∼15°C. The most abundant soil types at the study site are Cambisols that developed on paleozoic metamorphosed schists and sandstones. Soils are heterogeneous but usually nutrient-poor (thick organic layers can harbor locally high nutrient stocks, but often these are only slowly bioavailable) [41]. Estimates of total annual nutrient depositions (based on the monitoring of bulk and dry deposition between 1998 and 2010) range from 14–45 kg N ha^−1^ and 0.4–4.9 kg P ha^−1^ for the study area.

All necessary permits were required for the described field studies.

### Experimental design

A full-factorial nutrient manipulation experiment (NUMEX) was conducted in 16 plots of 400 m^2^ (20 m×20 m) consisting of four treatments (N, P, N+P, control) with four replicates in a stratified random design in four blocks at 2020–2120m a.s.l. (Fig. S1). Minimum distance between two plots was 10 m.

Nitrogen and phosphorus were added at an annual rate of 50 kg N ha^−1^ as urea and 10 kg P ha^−1^ as monosodium phosphate. The fertilizer was dispersed homogeneously over the plots with two application dates per year (January 26 and July 26) starting in 2008.

The dominant tree species in the NUMEX plots, making up about one quarter of all stems (dbh≥10 cm), was *Graffenrieda emarginata* Ruiz & Pav. (Melastomataceae); other frequently found species were *Myrcia* sp. nov. (Myrtaceae), *Alchornea lojaensis* Secco and *Hieronyma fendleri* Briq. (both Euphorbiaceae). The mean number of trees, mean tree diameter and stem basal area per plot (pre-fertilization survey of trees ≥10 cm dbh) were 45.7, 15.0 cm and 0.91 m^2^, respectively. Average stand height was 12 to 14 m.

### Organic layer nutrient pools

In April 2009, the soil organic layer (including the Oi, Oe, and Oa horizons) was sampled with a 0.2×0.2 m frame to the depth of the organic layer/mineral soil boundary at five randomly selected points within each permanent plot. Samples were dried to constant mass at 40°C.

The organic horizons were separated from the underlying mineral soil at the point where bulk density abruptly increases from <0.2 g cm^3^ in the organic layers to >1 g cm^3^ in the mineral soil [41].

The N concentrations in the ground samples were determined with a CHNS-analyzer (Vario EL Cube, Elementar Analysensysteme GmbH). After microwave digestion with HNO_3_ (Mars 5 Xpress, CEM Corporation, Matthews, NC), the total phosphate concentration was detected photometrically (Continuous Flow Analyser; Bran+Luebbe GmbH, Norderstedt, Germany). Soil bulk density was determined with two additional samples per plot which were dried at 105°C for 24 h. Detailed results are shown in Table S1.

### Microbial biomass

In May 2009, three samples per plot were collected in the upper organic layer to a depth of 5 cm using a metal corer (5 cm diameter). The upper litter layer (1–2 cm) was removed and the three samples were pooled and stored at 5°C. Before measurements, roots >2 mm were removed from the soil and the remaining material was chopped to pieces of <25 mm^2^, homogenized and pre-incubated at 20°C for five days.

Respiration of soil microorganisms was measured as O_2_ consumption using an automated electrolytic O_2_ microcompensation apparatus [72]. Respiration was measured at hourly intervals at 22°C for 24 h. Basal respiration (BR) of microorganisms was calculated as the mean oxygen consumption rates of hours 14–24 after the start of the measurements without addition of substrate. Microbial biomass carbon (C_mic_) was calculated from substrate induced respiration measuring the respiratory response to D-glucose which activates the metabolism of living microorganisms in the soil [73]. After adding Glucose (80 mg g^−1^ dry mass in 300 µl deionized water) the mean of the lowest three readings within the first 5–10 h was taken as maximum initial respiratory response (MIRR; µg O_2_ h^−1^ g^−1^ soil dry mass). Microbial biomass (µg C g^−1^ soil dry mass) was calculated as 38×MIRR [74].

### Net rates of nitrogen cycling in the soil

Net rates of N cycling in the soil were measured in October 2008 using the buried bag method [75]. In each plot a soil sample was taken from 0 to 5 cm depth. One subsample was extracted immediately in the field with 0.5 mol L^−1^ K_2_SO_4_ to determine initial NH_4_^+^ and NO_3_^−^ levels (T_0_). The other sample was put into a plastic bag, reburied in the soil, incubated for ten days and afterwards extracted with 0.5 mol L^−1^ K_2_SO_4_ (T_1_). The plastic bag was closed with a rubber band to prevent rain coming in but not too tight to permit air exchange. Net N mineralization and nitrification rates were calculated as the difference between T_1_- and T_0_- NH_4_^+^ and NO_3_^−^ concentration.

### Trace gas measurements

Nitrous oxide was measured monthly using static vented chambers. Four permanent chamber bases made of polyvinyl chloride (area 0.04 m^2^, height 0.25 m, ∼0.02 m inserted into the soil) were randomly placed in four of six subplots per plot at least four weeks before the first measurement, resulting in 16 chamber bases per block and 48 in total. Four gas samples (100 ml each) were removed at 2, 14, 26 and 38 min after chamber closure with an acrylonitrile butadiene styrene (ABS) lid and stored in pre-evacuated glass containers (60 mL) with stopcocks [76]. Gas samples were transported to the laboratory in Loja (Ecuador) within two days and analyzed using a gas chromatograph (Shimadzu GC-14B, Duisburg, Germany) equipped with an electron capture detector (ECD) and an autosampler [77]. Gas concentrations were determined by comparison of integrated peak areas of samples to standard gases (320, 501, 1001 and 3003 ppb N_2_O; Deuste Steininger GmbH, Mühlhausen, Germany). Gas fluxes were calculated from the linear increase of gas concentration in the chamber vs. time, and were adjusted for air temperature and atmospheric pressure [76]. Zero fluxes were included.

### Litterfall

Six litter traps (each 0.36 m^2^ in surface area positioned 1 m above ground) were randomly placed in each plot. The litterfall was collected every four weeks starting on November 6^th^, 2007. The collected samples were oven-dried at 60°C before determining the dry weight.

### Leaf morphology

Leaf samples from sun-exposed branches of each of 4–5 trees per treatment from four common species (*Alchornea lojaensis*, *Graffenrieda emarginata*, *Hieronyma fendleri* and *Myrcia* sp. nov.) were collected in January 2009 to quantify changes in leaf morphology and foliar nutrient concentrations one year after the onset of the experiment. For each sample 10–25 fresh leaves were scanned using a flat bed scanner (CanonScan LIDE 30, Canon). The images were analyzed subsequently with the WinFolia 2001a software (Regent Instruments Inc., Quebec, Canada) for calculation of leaf area. The leaves were then dried at 60°C to constant mass. Specific leaf area (SLA) was calculated as the ratio of leaf area and leaf dry weight.

### Foliar and litter nutrient contents

The concentrations of total C and N in leaf and litter mass were determined with a C/N elemental analyzer (Vario EL III, elementar, Hanau, Germany). The concentrations of total P were analyzed using an Inductively Coupled Plasma Analyzer (Optima 5300DV ICP-OES, Perkin Elmer) after digesting the samples with concentrated HNO_3_.

### Throughfall and soil solutions

Throughfall was collected with 20 randomly distributed, fixed-positioned funnel gauges in each plot. The volume of throughfall water was measured in the field with a graduated cylinder, and the samples were then bulked according to their relative volume to result in a single sample per plot per collecting date.

Litter leachate was collected using three zero-tension lysimeters per plot, which consisted of plastic boxes with a collecting surface area (polyethylene net) of 0.15 m×0.15 m, installed below the organic layer. All collected litter leachate samples of a plot were bulked to yield a single sample per plot per collecting date.

In each plot, mineral soil solution was collected using three suction lysimeters (ceramic suction cups with 1 µm pore size) at 0.15 and 0.30 m depth, installed so that bulking of the soil solution per soil depth occurred *in situ*.

Throughfall, litter leachate and mineral soil solutions were sampled fortnightly. After collecting the mineral soil solution, a vacuum was applied to the suction lysimeters in order to collect sufficient sample for the next sampling period.

After field collection, throughfall, litter leachate, and soil solution samples were transported to our field laboratory where an aliquot was filtered (ashless filters with pore size 4–7 µm, folded filter type 389; Munktell & Filtrak GmbH, Bärenstein, Germany) and frozen until transport to Germany for further analysis. Samples were analyzed for concentrations of NH_4_^+^, NO_3_^−^, total dissolved N, and total dissolved P using continuous flow analysis (CFA, Bran+Luebbe GmbH, Norderstedt, Germany). Dissolved organic nitrogen concentrations were calculated as the difference between total dissolved N and the sum of total inorganic nitrogen (NH_4_^+^+NO_3_^−^), assuming that NO_2_^−^ concentrations were negligible.

### Leaf area index

The LAI was quantified in the plots with two LAI-2000 plant canopy analyzers (LI-COR Inc., Lincoln, NE, USA). The LAI measurements were conducted in the remote mode, i.e. by synchronous readings below the canopy at 2 m height above the forest floor and in a nearby open area (“above-canopy” reading) using two devices. One measurement was done above each litter trap and a second at the same time outside the forest. Measurements were done in January 2008 (before 1^st^ fertilization) and in January 2009 (one year after the 1^st^ fertilization).

### Stem diameter growth and basal area growth per plot

The stem diameter growth of all trees present with a dbh≥10 cm was monitored in the 16 plots every six weeks with permanent girth-increment tapes (D1 dendrometer, UMS, Munich; 713 stems in total). The cumulative increase in plot basal area per year was calculated as the sum of all tree basal area increments in a plot between February 15^th^, 2008 (after the first fertilization) and January 19^th^, 2009.

### Fine root biomass

For measuring fine root biomass, we took six root samples per plot to a depth of 20 cm using a soil corer of 3.5 cm in diameter in January 2009. The soil samples were transferred to plastic bags and transported to the laboratory, where processing of the stored samples (4°C) took place within six weeks. In the lab, the samples were soaked in water and cleaned from soil residues using a sieve with a mesh size of 0.25 mm. Only fine roots (roots <2 mm in diameter) of trees were considered for analysis. Live fine roots (biomass) were separated from dead rootlets (necromass) under the stereomicroscope based on color, root elasticity, and the degree of cohesion of cortex, periderm and stele [78]–[79]. The fine root biomass of each sample was dried at 70°C for 48 h and weighed.

### Mycorrhiza

Root colonization by arbuscular mycorrhizal fungi was measured at 200× magnification following clearing and staining with 0.05% Trypan Blue according to [80], additionally including fungal structures as defined in [81].

### Statistical analyses

The effects of N and/or P addition on the various investigated parameters were expressed by a response ratio metric (RR_X_ = *ln* (measured value in nutrient addition treatment/measured value in the control)) [8] in order to compare the response of plant- and soil-related state variables or flux parameters in relative terms. Non-transformed data are shown in Table S2.

Effects of the addition of N and/or P on individual parameters were analyzed using linear mixed models (package lme4, R version 2.13.0) [82]. We included the fertilization treatments as fixed effects and the factor “block” as a random factor in the models, since in most parameters, samples were nested within plots. P-values for the fixed effects were calculated with the “cftest” function of the package “multcomp” (R version 2.13.0) [82].

## Supporting Information

Figure S1 **Location of the study area in southern Ecuador and outline of the Ecuadorian Nutrient Manipulation EXperiment (NUMEX).**
(DOC)

Table S1 **Soil nutrient status of the experimental plots in July 2007 prior to the first fertilization.**
(DOC)

Table S2 **Ranges and means of all parameters shown in** **Figures 1** **–** [Fig pone-0047128-g002] [Fig pone-0047128-g003] [Fig pone-0047128-g004] **5** **.**
(DOC)

## References

[1] Galloway JN, Townsend AR, Erisman JW, Bekunda M, Cai Z, et al (2008) Transformation of the nitrogen cycle: recent trends, questions, and potential solutions. Science 320: 889–892. 18487183 10.1126/science.1136674

[2] Gruber N, Galloway N (2008) An earth-system perspective of the global nitrogen cycle. Nature 451: 293–296. 18202647 10.1038/nature06592

[3] Janssens IA, Dieleman W, Luyssaert S, Subke J-A, Reichstein M, et al (2010) Reduction of forest soil respiration in response to nitrogen deposition. Nat Geosci 3: 315–322.

[4] Mahowald NM, Artaxo P, Baker AR, Jickells TD, Okin GS, et al (2005) Impacts of biomass burning emissions and land use change on Amazonian atmospheric phosphorus cycling and deposition. Global Biogeochem Cycles 19: GB4030.

[5] Schlesinger WH (2009) On the fate of anthropogenic nitrogen. Proc Natl Acad Sci U S A 106: 203–208. 19118195 10.1073/pnas.0810193105 PMC2613040

[6] Tilman D, Fargione J, Wolff B, D'Antonio C, Dobson A, et al (2001) Forecasting agriculturally driven global environmental change. Science 292: 281–284. 11303102 10.1126/science.1057544

[7] Bobbink R, Hicks K, Galloway J, Spranger T, Alkemade R, et al (2010) Global assessment of nitrogen deposition effects on terrestrial plant diversity: a synthesis. Ecol Appl 20: 30–59. 20349829 10.1890/08-1140.1

[8] Elser JJ, Bracken MES, Cleland EE, Gruner DS, Harpole WS, et al (2007) Global analysis of nitrogen and phosphorus limitation of primary producers in freshwater, marine and terrestrial ecosystems. Ecol Lett 10: 1135–1142. 17922835 10.1111/j.1461-0248.2007.01113.x

[9] Reay DS, Dentener F, Smith P, Grace J, Feely RA (2008) Global nitrogen deposition and carbon sinks. Nat Geosci 1: 430–437.

[10] Harpole WS, Ngai JT, Cleland EE, Seabloom EW, Borer ET, et al (2011) Nutrient co-limitation of primary producer communities. Ecol Lett 14: 852–862. 21749598 10.1111/j.1461-0248.2011.01651.x

[11] Hedin LO, Brookshire ENJ, Menge DNL, Barron A (2009) The nitrogen paradox in tropical forest ecosystems. Annu Rev Ecol Evol Syst 40: 613–635.

[12] LeBauer DS, Treseder KK (2008) Nitrogen limitation of net primary productivity in terrestrial ecosystems is globally distributed. Ecology 89: 371–379. 18409427 10.1890/06-2057.1

[13] Vitousek PM, Porder S, Houlton BZ, Chadwick OA (2010) Terrestrial phosphorus limitation: mechanisms, implications, and nitrogen-phosphorus interactions. Ecol Appl 20: 5–15. 20349827 10.1890/08-0127.1

[14] Xia J, Wan S (2008) Global response patterns of terrestrial plant species to nitrogen addition. New Phytol 179: 428–439. 19086179 10.1111/j.1469-8137.2008.02488.x

[15] Bedison JE, McNeill BE (2009) Is the growth of temperate forest trees enhanced along an ambient nitrogen deposition gradient? Ecology 90: 1736–1742. 19694123 10.1890/08-0792.1

[16] Wright RF, Rasmussen L (1998) Introduction to the NITREX and EXMAN projects. For Ecol Manage 101: 1–7.

[17] Stevens CJ, Dise NB, Mountford JO, Gowing DJ (2004) Impact of nitrogen deposition on the species richness of grasslands. Science 303: 1876–1879. 15031507 10.1126/science.1094678

[18] Högberg P, Fan H, Quist M, Binkley D, Tamm CO (2006) Tree growth and soil acidification in response to 30 years of experimental nitrogen loading on boreal forest. Glob Chang Biol 12: 489–499.

[19] Bouwman L, Goldewijka KK, Van Der Hoek KW, Beusena AHW, Van Vuurena DP, et al (in press) Exploring global changes in nitrogen and phosphorus cycles in agriculture induced by livestock production over the 1900–2050 period. Proc Natl Acad Sci U S A doi: 10.1073/pnas.1012878108. 10.1073/pnas.1012878108 PMC3876211 21576477

[20] Phoenix G, Hicks WK, Cinderby S, Kuylenstierna JCI, Stocks WD, et al (2006) Atmospheric nitrogen deposition in world biodiversity hotspots: the need for a greater global perspective in assessing N deposition impacts. Glob Chang Biol 12: 470–476.

[21] Tamatamah RA, Hecky RE, Duthie HD (2005) The atmospheric deposition of phosphorus in Lake Victoria (East Africa). Biogeochemistry 73: 325–344.

[22] Okin GS, Mahowald N, Chadwick OA, Artaxo P (2004) Impact of desert dust on the biogeochemistry of phosphorus in terrestrial ecosystems. Global Biogeochem Cycles 18: 1–14.

[23] Pett-Ridge JC (2009) Contributions of dust to phosphorus cycling in tropical forests of the Luquillo Mountains, Puerto Rico. Biogeochemistry 94: 63–80.

[24] Fabian P, Kohlpaintner M, Rollenbeck R (2005) Biomass burning in the Amazon – fertilizer for the mountaineous rain forest in Ecuador. Environ Sci Pollut Res Int 12: 290–296. 16206723 10.1065/espr2005.07.272

[25] Boy J, Rollenbeck R, Valarezo C, Wilcke W (2008) Amazonian biomass burning-derived acid and nutrient deposition in the north Andean montane forest of Ecuador. Global Biogeochem Cycles 22: GB4011.

[26] Matson PA, McDowell WH, Townsend AR, Vitousek PM (1999) The globalization of N deposition: ecosystem consequences in tropical environments. Biogeochemistry 46: 67–83.

[27] Corre MD, Veldkamp E, Arnold J, Wright SJ (2010) Impact of elevated N input on N cycling and retention of soils under old-growth lowland and montane forests in Panama. Ecology 91: 1715–1729. 20583713 10.1890/09-0274.1

[28] Hall SJ, Matson PA (2003) Nutrient status of tropical rain forests influences soil N dynamics after N addition. Ecol Monogr 73: 107–129.

[29] Lewis SL, Malhi Y, Phillips OL (2004) Fingerprinting the impacts of global change on tropical forests. Phil Trans R Soc Lond B Biol Sci 359: 437–462. 15212095 10.1098/rstb.2003.1432 PMC1693339

[30] Li Y, Xu M, Zou X (2006) Effects of nutrient additions on ecosystem carbon cycle in a Puerto Rican tropical wet forest. Glob Chang Biol 12: 284–293.

[31] Wright SJ (2005) Tropical forests in a changing environment. Trends Ecol Evol 20: 553–560. 16701434 10.1016/j.tree.2005.07.009

[32] Adamek M, Corre MD, Hölscher D (2009) Early effect of elevated nitrogen input on above-ground net primary production of a lower montane rain forest, Panama. J Trop Ecol 25: 637–647.

[33] Cavelier J, Tanner E, Santamaria J (2000) Effect of water, temperature and fertilizers on soil nitrogen net transformations and tree growth in an elfin cloud forest of Colombia. J Trop Ecol 83–99.

[34] Tanner EVJ, Kapos V, Freskos S, Healey JR, Theobald AM (1990) Nitrogen and phosphorus fertilization of Jamaican montane forest trees. J Trop Ecol 6: 231–238.

[35] Tanner EVJ, Kapos V, Franco W (1992) Nitrogen and phosphorus fertilization effects on Venezuelan montane forest trunk growth and litterfall. Ecology 73: 78–86.

[36] Mirmanto E, Proctor J, Green J, Nagy L (1999) Suriantata (1999) Effects of nitrogen and phosphorus fertilization in a lowland evergreen rainforest. Phil Trans R Soc Lond B Biol Sci 354: 1825–1829. 11605625 10.1098/rstb.1999.0524 PMC1692691

[37] Nomura N, Kikuzawa K (2003) Productive phenology of tropical montane forests: Fertilization experiments along a moisture gradient. Ecol Res 18: 573–586.

[38] Ostertag R (2010) Foliar nitrogen and phosphorus accumulation responses after fertilization: an example from nutrient-limited Hawaiian forests. Plant Soil 334: 85–98.

[39] Vitousek PM, Farrington H (1997) Nutrient limitation and soil development: Experimental test of a biogeochemical theory. Biogeochemistry 37: 63–75.

[40] Wright SJ, Yavitt JB, Wurzburger N, Turner BL, Tanner EVJ, et al (2011) Potassium, phosphorus, or nitrogen limit root allocation, tree growth, or litter production in a lowland tropical forest. Ecology 92: 1616–1625. 21905428 10.1890/10-1558.1

[41] Wilcke W, Yasin S, Abramowski U, Valarezo C, Zech W (2002) Nutrient storage and turnover in organic layers under tropical montane rain forest in Ecuador. Europ J Soil Sci 53: 15–27.

[42] Treseder KK (2008) Nitrogen additions and microbial biomass: a meta-analysis of ecosystem studies. Ecol Lett 11: 1111–1120. 18673384 10.1111/j.1461-0248.2008.01230.x

[43] Fog K (1988) The effect of added nitrogen on the rate of decomposition of organic matter. Biol Rev 63: 433–462.

[44] Lu M, Yang Y, Luo Y, Fang C, Zhou X, et al (2011) Responses of ecosystem nitrogen cycle to nitrogen addition: a meta-analysis. New Phytol 189: 1040–1050. 21138438 10.1111/j.1469-8137.2010.03563.x

[45] Lohse KA, Matson P (2005) Consequences of nitrogen additions for soil losses from wet tropical forests. Ecol Appl 15 (5) 1629–1648.

[46] Matzner E, Zuber T, Lischeid G (2004) Response of soil solution chemistry and solute fluxes to changing deposition rates. In: Matzner E, editor. Biogeochemstry of forested catchments in a changing environment - A German case study. Ecological Studies Vol 172. Berlin: Springer. pp. 339–360.

[47] Goller R, Wilcke W, Fleischbein K, Valarezo C, Zech W (2006) Dissolved nitrogen, phosphorus, and sulfur forms in the ecosystem fluxes of a montane forest in Ecuador. Biogeochemistry 77: 57–89.

[48] Wullaert H, Homeier J, Valarezo C, Wilcke W (2010) Response of the N and P cycles of an old-growth montane forest in Ecuador to experimental low-level N and P amendments. For Ecol Manage 260: 1434–1445.

[49] Wieder WR, Cleveland CC, Townsend AR (2009) Controls over leaf litter decomposition in wet tropical forests. Ecology 90: 3333–3341. 20120803 10.1890/08-2294.1

[50] Cleveland CC, Townsend AR (2006) Nutrient additions to a tropical rain forest drive substantial soil carbon dioxide losses to the atmosphere. Proc Natl Acad Sci U S A 103: 10316–10321. 16793925 10.1073/pnas.0600989103 PMC1502455

[51] Treseder KK, Vitousek PM (2001) Effects of soil nutrient availability on investment in acquisition of N and P in Hawaiian rain forests. Ecology 82: 946–954.

[52] Gress SE, Nichols TD, Northcraft CC, Peterjohn W (2007) Nutrient limitation in soils exhibiting differing nitrogen availabilities: what lies beyond nitrogen saturation? Ecology 88: 119–130. 17489460 10.1890/0012-9658(2007)88[119:nlised]2.0.co;2

[53] Olander LP, Vitousek PM (2000) Regulation of soil phosphatase and chitinase activity by N and P availability. Biogeochemistry 49: 175–190.

[54] Poorter L, Bongers F (2006) Leaf traits are good predictors of plant performance across 53 rain forest species. Ecology 87: 1733–1743. 16922323 10.1890/0012-9658(2006)87[1733:ltagpo]2.0.co;2

[55] Vitousek PM, Walker LR, Whiteaker LD, Matson PA (1993) Nutrient limitations to plant growth during primary succession in Hawaii Volcanoes National Park. Biogeochemistry 23: 197–215.

[56] Güsewell S, Gessner MO (2009) N∶P ratios influence litter decomposition and colonization by fungi and bacteria in microcosms. Funct Ecol 23: 211–219.

[57] Cusack DF, Silver WL, Torn MS, McDowell WH (2011) Effects of nitrogen additions on above- and belowground carbon dynamics in two tropical forests. Biogeochemistry 104: 203–225.

[58] Gower ST, Vitousek PM (1989) Effects of nutrient amendments on fine root biomass in a primary successional forest in Hawai'i. Oecologia 81: 566–568. 28312655 10.1007/BF00378970

[59] Nadelhoffer KJ (2000) The potential effects of nitrogen deposition on fine-root production in forest ecosystems. New Phytol 147: 131–139.

[60] Yuan ZY, Chen HYH (2012) A global analysis of fine root production as affected by soil nitrogen and phosphorus. Proc R Soc Lond B Biol Sci 279: 3796–3802. 10.1098/rspb.2012.0955 PMC3415909 22764168

[61] Johnson NC, Rowland DL, Corkidi L, Allen EB (2008) Plant winners and losers during grassland N-eutrophication differ in biomass allocation and mycorrhizas. Ecology 89: 2868–2878. 18959324 10.1890/07-1394.1

[62] Treseder KK (2004) A meta-analysis of mycorrhizal responses to nitrogen, phosphorus, and atmospheric CO_2_ in field studies. New Phytol 164: 347–355. 10.1111/j.1469-8137.2004.01159.x 33873547

[63] Powell JR, Parrent JL, Hart MM, Klironomos JN, Rillig MC, et al (2009) Phylogenetic trait conservatism and the evolution of functional tradeoffs in arbuscular mycorrhizal fungi. Proc R Soc Lond B Biol Sci 276: 4237–4245. 10.1098/rspb.2009.1015 PMC2821337 19740877

[64] Vitousek PM (1984) Litterfall, nutrient cycling, and nutrient limitation in tropical forests. Ecology 65: 285–298.

[65] Kobe RK, Lepczyk CA, Iyer M (2005) Resorption efficiency decreases with increasing green leaf nutrients in a global data set. Ecology 86: 2780–2792.

[66] Güsewell S (2004) N∶P ratios in terrestrial plants: variation and functional significance. New Phytol 164: 243–266. 10.1111/j.1469-8137.2004.01192.x 33873556

[67] Lu X, Mo J, Gilliam FS, Zhou G, Fang Y (2010) Effects of experimental nitrogen additions on plant diversity in old-growth tropical forest. Glob Chang Biol 16: 2688–2700.

[68] Townsend AR, Cleveland CC, Asner GP, Bustamente MMC (2007) Controls over foliar N∶P rations in tropical rain forests. Ecology 88: 107–118. 17489459 10.1890/0012-9658(2007)88[107:cofnri]2.0.co;2

[69] Wright IJ, Reich PB, Westoby M, Ackerly DD, Baruch Z, et al (2004) The worldwide leaf economics spectrum. Nature 428: 821–827. 15103368 10.1038/nature02403

[70] Beck E, Bendix J, Kottke I, Makeschin F, Mosandl R, editors (2008) Gradients in a tropical mountain ecosystem of Ecuador. Ecological Studies Vol. 198. Berlin: Springer. 525 p.

[71] Homeier J, Breckle S-W Günter S, Rollenbeck RT, Leuschner C (2010) Tree diversity, forest structure and productivity along altitudinal and topographical gradients in a species-rich Ecuadorian montane rainforest. Biotropica 42: 140–148.

[72] Scheu S (1992) Automated measurement of the respiratory response of soil microcompartments: Active microbial biomass in earthworm faeces. Soil Biol Biochem 24: 1113–1118.

[73] Anderson JJPE, Domsch KH (1978) A physiological method for the quantitative measurement of microbial biomass in soil. Soil Biol Biochem 10: 215–221.

[74] Beck T, Joergensen RG, Kandeler E, Makeschin F, Nuss E, et al (1997) An inter-laboratory comparison of ten different ways of measuring soil microbial biomass C. Soil Biol Biochem 29: 1023–1032.

[75] Hart SC, Stark JM, Davidson EA & Firestone MK (1994) Nitrogen mineralization, immobilization and nitrification. In: Methods of Analysis Part 2. Microbiological and Biochemical Properties. Madison: Soil Science Society of America Book Series. pp 985–1018.

[76] Koehler B, Corre MD, Veldkamp E, Wullaert H, Wright SJ (2009) Immediate and long-term nitrogen oxide emissions from tropical forest soils exposed to elevated nitrogen input. Glob Chang Biol 15: 2049–2066.

[77] Loftfield N, Flessa H, Augustin J, Beese F (1997) Automated gas chromatographic system for rapid analysis of the atmospheric trace gases methane, carbon dioxide, and nitrous oxide. J Environ Qual 26: 560–564.

[78] Persson H (1978) Root dynamics in a young Scots pine stand in Central Sweden. Oikos 30: 508–519.

[79] Leuschner C, Hertel D, Coners H, Büttner V (2001) Root competition between beech and oak: a hypothesis. Oecologia 126: 276–284. 28547627 10.1007/s004420000507

[80] Rillig MC, Allen MF, Klironomos JN, Chiariello NR, Field CB (1998) Plant species-specific changes in root-inhabiting fungi in a California annual grassland: responses to elevated CO2 and nutrients. Oecologia 113: 252–259. 28308205 10.1007/s004420050376

[81] Beck A, Haug I, Oberwinkler F, Kottke I (2007) Structural characterization and molecular identification of arbuscular mycorrhiza morphotypes of Alzatea verticillata (Alzateaceae), a prominent tree in the tropical mountain rain forest of South Ecuador. Mycorrhiza 17: 607–625. 17653774 10.1007/s00572-007-0139-0

[82] R Development Core Team (2011) R: A language and environment for statistical computing. Vienna: R Foundation for Statistical Computing.

