# Supplementary material for: Tropical Andean Forests Are Highly Susceptible to Nutrient Inputs—Rapid Effects of Experimental N and P Addition to an Ecuadorian Montane Forest
Source: PLoS One. 2012 Oct 10;7(10):e47128. doi: 10.1371/journal.pone.0047128 (PMC3468540; doi:10.1371/journal.pone.0047128)
Supplement: Figure S1 — Location of the study area in southern Ecuador and outline of the Ecuadorian Nutrient Manipulation EXperiment (NUMEX). (DOC) [file pone.0047128.s001.doc]

**Supporting Information Figure S1**

**A**

**B**


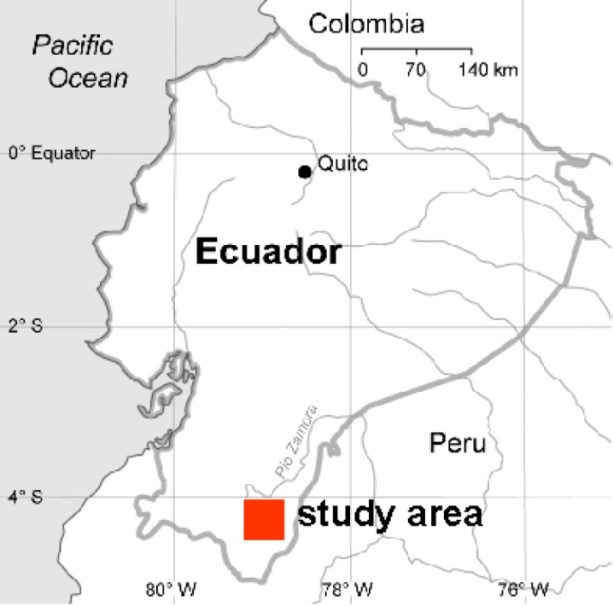

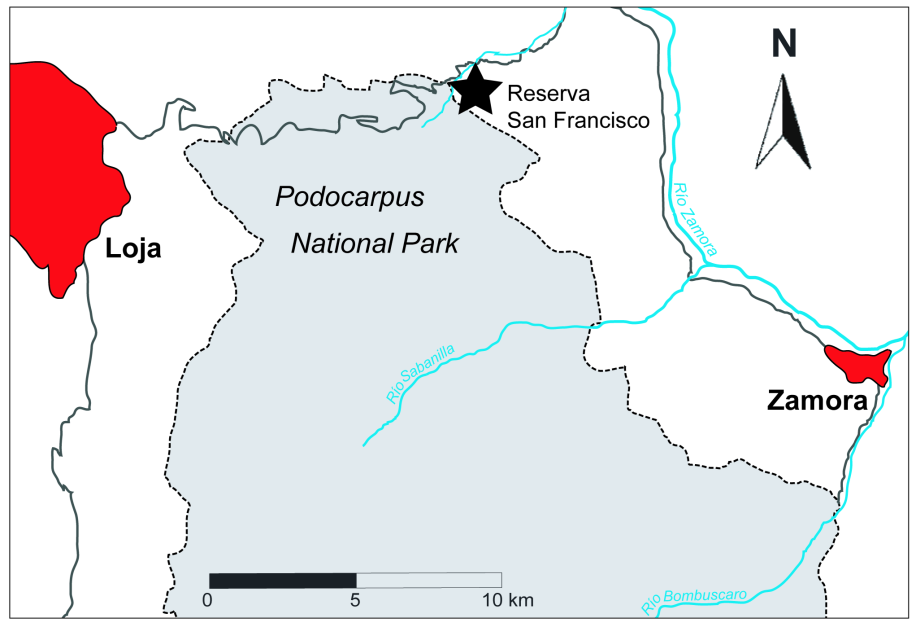


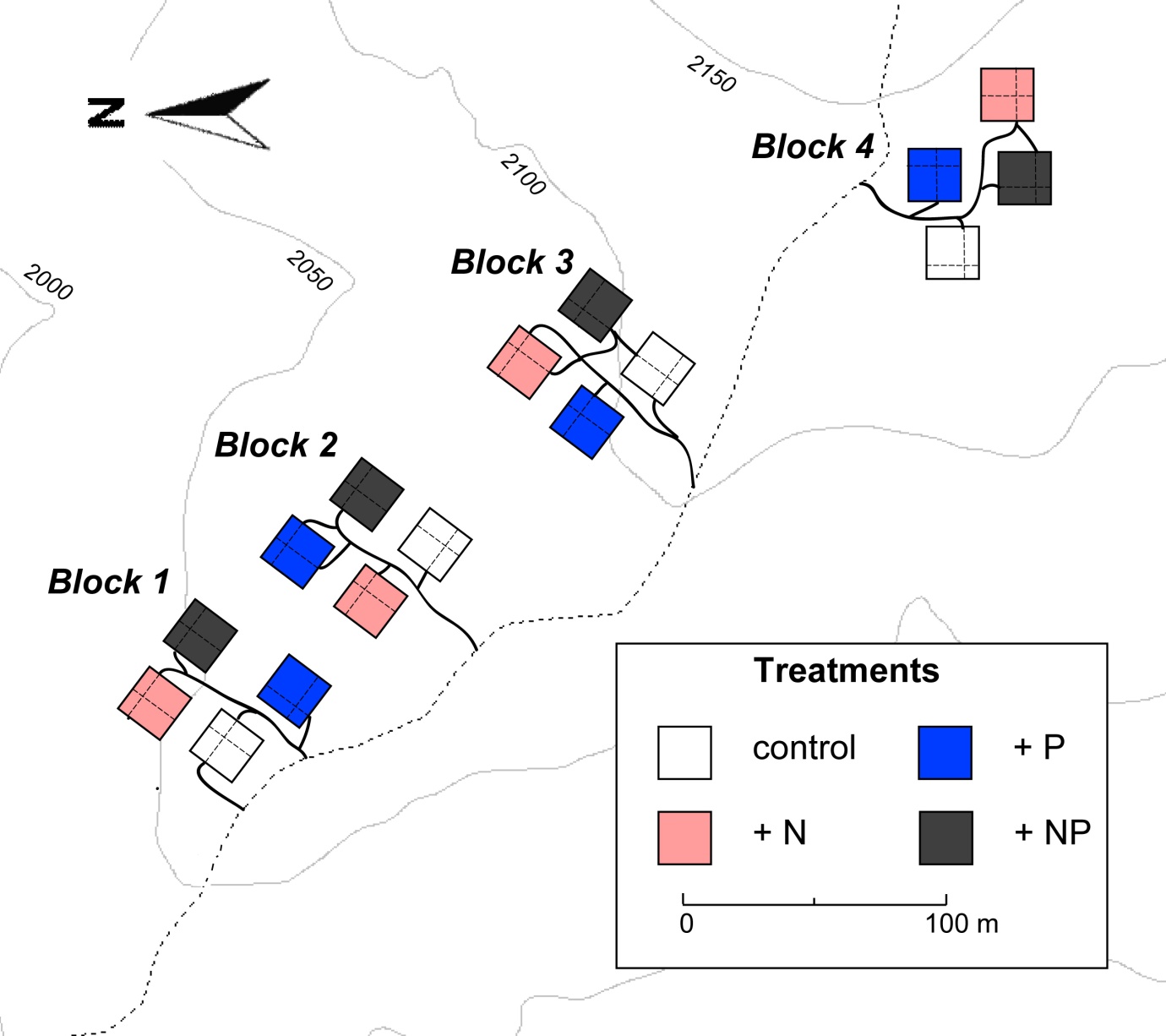


**C**

**Figure S1.** **Location of the study area in southern Ecuador and outline of the Ecuadorian Nutrient Manipulation EXperiment (NUMEX). A.** and **B.** Location of the study area in southern Ecuador. **C.** Outline of the Ecuadorian Nutrient Manipulation EXperiment (NUMEX). The study was conducted in 16 plots à 400 m² (20m x 20m) consisting of four treatments (control, N, P, N+P); the replicates were randomly arranged in four blocks at ~ 2100 m a.s.l..
