# Supplementary material for: Tropical Andean Forests Are Highly Susceptible to Nutrient Inputs—Rapid Effects of Experimental N and P Addition to an Ecuadorian Montane Forest
Source: PLoS One. 2012 Oct 10;7(10):e47128. doi: 10.1371/journal.pone.0047128 (PMC3468540; doi:10.1371/journal.pone.0047128)
Supplement: Table S1 — Soil nutrient status of the experimental plots in July 2007 prior to the first fertilization. (DOC) [file pone.0047128.s002.doc]

**Supporting Information Table 1**

| **Table S1. Soil nutrient status of the experimental plots in July 2007 before the first fertilization.** | | | | | |
| --- | --- | --- | --- | --- | --- |
|  |  | **Control** | **+N** | **+P** | **+NP** |
|  |  |  |  |  |  |
| **Org. layer depth (cm)** | O-horizon | 28.8 ± 10.8 | 26.5 ± 3.9 | 23.8 ± 4.5 | 35.0 ± 4.4 |
|  |  |  |  |  |  |
| **pH (H_2_O)** | O-horizon | 3.25 ± 0.25 | 3.33 ± 0.17 | 3.28 ± 0.08 | 3.36 ± 0.09 |
|  | A-horizon | 3.66 ± 0.12 | 3.69 ± 0.09 | 3.63 ± 0.06 | 3.67 ± 0.03 |
|  | B-horizon | 4.05 ± 0.20 | 3.95 ± 0.19 | 3.95 ± 0.19 | 3.93 ± 0.21 |
|  |  |  |  |  |  |
| **Total C** | O-horizon | 42.90 ± 3.14 | 42.90 ± 1.13 | 43.78 ± 1.78 | 43.47 ± 0.96 |
| **(%)** | A-horizon | 1.05 ± 0.24 | 1.20 ± 0.26 | 1.56 ± 0.28 | 1.20 ± 0.40 |
|  | B-horizon | 0.67 ± 0.29 | 0.71 ± 0.31 | 0.52 ± 0.02 | 0.95 ± 0.39 |
|  |  |  |  |  |  |
| **Total N** | O-horizon | 2.03 ± 0.14 | 2.11 ± 0.20 | 2.03 ± 0.18 | 2.21 ± 0.09 |
| **(%)** | A-horizon | 0.06 ± 0.02 | 0.08 ± 0.02 | 0.08 ± 0.02 | 0.08 ± 0.03 |
|  | B-horizon | 0.04 ± 0.01 | 0.05 ± 0.02 | 0.04 ± 0.01 | 0.07 ± 0.03 |
|  |  |  |  |  |  |
| **C:N ratio** | O-horizon | 21.1 ±1.4 | 20.5 ±1.4 | 21.7 ± 1.5 | 19.7 ± 0.5 |
|  | A-horizon | 17.0 ± 1.6 | 16.0 ± 1.8 | 19.5 ± 2.6 | 15.4 ± 2.6 |
|  | B-horizon | 16.2 ± 4.8 | 14.4 ± 0.2 | 14.9 ± 4.8 | 14.7 ± 5.4 |
|  |  |  |  |  |  |
| **Total P** | mineral topsoil |  |  |  |  |
| **(mg/g)** | (0-10cm) | 0.06 ± 0.01 | 0.07 ± 0.01 | 0.07 ± 0.02 | 0.06 ± 0.02 |
|  | (10-20cm) | 0.10 ± 0.03 | 0.09 ± 0.02 | 0.13 ± 0.05 | 0.12 ± 0.07 |
|  |  |  |  |  |  |
| **ECEC** | A-horizon | 3.0 ± 1.4 | 3.9 ± 0.8 | 3.5 ± 1.3 | 3.3 ± 0.8 |
| **(cmol c/kg)** | B-horizon | 3.2 ± 1.1 | 3.8 ± 0.5 | 3.5 ± 1.5 | 4.3 ± 1.3 |
|  |  |  |  |  |  |
| **Base** | A-horizon | 4.1 ± 3.1 | 3.7 ± 1.3 | 4.4 ± 2.6 | 3.4 ± 0.4 |
| **saturation (%)** | B-horizon | 1.8 ± 0.9 | 1.4 ± 0.8 | 1.6 ± 0.5 | 1.2 ± 0.7 |
| Shown are means (± SD) of the four plots per treatment (one composite sample per plots). | | | | | |
