# Supplementary material for: Tropical Andean Forests Are Highly Susceptible to Nutrient Inputs—Rapid Effects of Experimental N and P Addition to an Ecuadorian Montane Forest
Source: PLoS One. 2012 Oct 10;7(10):e47128. doi: 10.1371/journal.pone.0047128 (PMC3468540; doi:10.1371/journal.pone.0047128)
Supplement: Table S2 — Ranges and means of all parameters shown in Figures 1 – 5 . (DOC) [file pone.0047128.s003.doc]

**Supporting Information Table 2**

| **Table S2. Effects of nutrient addition on different ecosystem parameters (Figures 2 – 6) of a tropical montane forest ecosystem.** | | | | | |
| --- | --- | --- | --- | --- | --- |
|  | **Time of measurement** | **Control** | **+N** | **+P** | **+NP** |
|  |  |  |  |  |  |
| Org. layer N pool (Mg N ha^-1^) | April 2009 | 3.78 (2.96–4.40) | 3.45 (2.61–4.41) | 3.51 (2.50–4.66) | 4.29 (3.84–4.87) |
| Org. layer P pool (kg P ha^-1^) | April 2009 | 98.6 (78.8–109.5) | 92.2 (81.4–107.6) | 101.9 (81.4–131.2) | 125.6 (110.1–142.1) |
| Soil microbial biomass (µg C_mic_ g^-1^ soil dry mass) | May 2009 | 5881 (5826–5930) | 4699 (3747–5723) | 6178 (5826–6524) | 5991 (5573–6749) |
| Respiration of soil microorganisms (µl O_2_ mg C_mic_^-1^ h^-1^) | May 2009 | 5.07 (4.74–6.14) | 9.25 (7.26–15.2) | 5.78 (4.00–6.60) | 6.97 (5.03–10.83) |
| Net N min. (ng N cm^2^ h^-1^) | September 2008 | 23.4 (2.8–30.4) | 44.4 (15.5–67.3) | 11.2 (4.1–19.3) | 40.5 (28.0–55.5) |
| Net N nitrification (ng N cm^2^ h^-1^) | September 2008 | 0 (0–0) | -1.68 (-5.04–0) | 0 (0–0) | 7.5 (0–14.9) |
| N_2_O emission (kg N ha^-1^ yr^-1^) | Feb 2008 –Jan 2009 | 0.245 (0.206–0.306) | 0.390 (0.232–0.572) | 0.123 (0.009–0.250) | 0.427 (0.231–0.564) |
| NH4/NO3 ratio (org. layer percolate) | Feb 2008 –Jan 2009 | 15.7 (4.4–28.9) | 11.0 (1.6–33.6) | 16.5 (0.9–27.6) | 4.8 (1.5–6.8) |
| NH4/NO3 ratio (soil solution) | Feb 2008 –Jan 2009 | 8.9 (3.0–11.9) | 7.8 (0.9–13.7) | 6.9 (1.0–11.5) | 2.5 (1.1–4.3) |
| Litterfall N conc. (% N) | January 2009 | 0.870 (0.816–0.945) | 1.018 (0.943–1.182) | 0.926 (0.903–0.956) | 0.981 (0.924–1.064) |
| Litterfall P conc. (mg g^-1^) | January 2009 | 0.251 (0.176–0.425) | 0.275 (0.241–0.335) | 0.338 (0.311–0.365) | 0.338 (0.260–0.392) |
| Annual return of N with litterfall (kg N ha^-1^ yr^-1^) | Feb 2008 –Jan 2009 | 41.7 (33.5–54.5) | 53.0 (27.7–70.5) | 43.5 (32.9–45.7) | 54.2 (44.5–62.4) |
| Annual return of P with litterfall (kg P ha^-1^ yr^-1^) | Feb 2008 –Jan 2009 | 1.48 (1.14–2.18) | 1.85 (0.94–2.77) | 1.80 (1.18–2.33) | 1.95 (1.61–2.33) |
| Annual return of N with throughfall (kg N ha^-1^ yr^-1^) | Feb 2008 –Jan 2009 | 10.09 (8.15–12.44) | 11.47 (10.02–12.61) | 10.64 (10.10–11.29) | 10.92 (10.50–11.47) |
| Annual return of P with throughfall (kg P ha^-1^ yr^-1^) | Feb 2008 –Jan 2009 | 0.151 (0.098–0.233) | 0.158 (0.132–0.187) | 0.210 (0.140–0.283) | 0.290 (0.203–0.370) |
| Org. layer percolate N (kg N ha^-1^ yr^-1^) | Feb 2008 –Jan 2009 | 14.65 (12.53–18.08) | 22.51 (19.07–35.59) | 14.16 (12.68–15.14) | 18.37 (16.99–19.58) |
| Org. layer percolate P (kg P ha^-1^ yr^-1^) | Feb 2008 –Jan 2009 | 0.13 (0.09–0.19) | 0.20 (0.11–0.31) | 0.21 (0.10–0.44) | 0.24 (0.12–0.39) |
| Table S2: continued | | | | | |
|  | **Time of measurement** | **Control** | **+N** | **+P** | **+NP** |
|  |  |  |  |  |  |
| Min. soil solution N (kg N ha^-1^ yr^-1^) | Feb 2008 –Jan 2009 | 3.26 (2.08–4.88) | 3.92 (3.48–4.48) | 3.55 (2.72–4.46) | 3.70 (2.96–4.24) |
| Min. soil solution P (kg P ha^-1^ yr^-1^) | Feb 2008 –Jan 2009 | 0.03 (0.02–0.04) | 0.04 (0.03–0.06) | 0.02 (0.02–0.03) | 0.03 (0.02–0.06) |
| LAI change (%) | Jan 2008 – Jan 2009 | 2.6 (-5.5–14.5) | 10.9 (1.1–20.9) | 11.7 (-4.7–24.7) | 5.3 (-8.2–18.9) |
| Tree basal area increment (m² ha^-1^) | Feb 2008 –Jan 2009 | 0.111 (0.081–0.162) | 0.109 (0.073–0.147) | 0.120 (0.052–0.175) | 0.160 (0.109–0.222) |
| Leaf litter production (Mg ha^-1^ yr^-1^) | Feb 2008 –Jan 2009 | 3.46 (2.71–4.75) | 3.65 (2.63–4.90) | 3.28 (2.44–4.47) | 3.99 (3.43–4.42) |
| Fine root biomass (g m^-2^) | January 2009 | 443 (337–567) | 375 (277–474) | 320 (245–398) | 346 (180–434) |
| Fine root necromass (g m^-2^) | January 2009 | 426 (307–527) | 447 (370–578) | 607 (570–631) | 441 (423–468) |
| Mycorrhiza colonization (%) | January 2009 | 53.3 (41.5–70.7) | 49.9 (34.6–66.4) | 42.8 (10.2–63.0) | 52.6 (39.4–59.4) |
| N use efficiency (g g^-1^ N) | January 2009 | 117 (106–124) | 100 (86–107) | 110 (106–114) | 104 (97–109) |
| P use efficiency (g g^-1^ P) | January 2009 | 4751 (2441–5895) | 4181 (3056–4929) | 3505 (2954–4200) | 3388 (2577–4306) |
| Shown are means and ranges (in parentheses) of the four replicates (plots) per treatment. The nutrient manipulation experiment started in January 2008. | | | | | |
